# Cut-off optimization for ^13^C-urea breath test in a community-based trial by mathematic, histology and serology approach

**DOI:** 10.1038/s41598-017-02180-7

**Published:** 2017-05-18

**Authors:** Zhe-Xuan Li, Lei-Lei Huang, Cong Liu, Luca Formichella, Yang Zhang, Yu-Mei Wang, Lian Zhang, Jun-Ling Ma, Wei-Dong Liu, Kurt Ulm, Jian-Xi Wang, Lei Zhang, Monther Bajbouj, Ming Li, Michael Vieth, Michael Quante, Tong Zhou, Le-Hua Wang, Stepan Suchanek, Erwin Soutschek, Roland Schmid, Meinhard Classen, Wei-Cheng You, Markus Gerhard, Kai-Feng Pan

**Affiliations:** 1 Key Laboratory of Carcinogenesis and Translational Research (Ministry of Education/Beijing), Department of Cancer Epidemiology, Peking University Cancer Hospital & Institute, 52 Fu-cheng Road, Hai-dian District, Beijing, 100142 China; 2 Technische Universität München, Klinikum rechts der Isar, Trogerstr. 30, 81675 Munich, Germany; 3 Healthy Bureau of Linqu County, Shandong, China; 4 Institute of Pathology, Klinikum Bayreuth, Preuschwitzer Str. 101, 95445 Bayreuth, Germany; 5 Charles University, Central Military Hospital Prague, Ovocný trh 3-5, Prague, 11636 Czech Republic; 6 Mikrogen GmbH, Floriansbogen 2-4, Neuried, Munich 82061 Germany; 7 International Digestive Cancer Alliance, 81541 Munich, Germany; 8 German Centre of Infection Research, partner site Munich, Munich, Germany

## Abstract

The performance of diagnostic tests in intervention trials of *Helicobacter pylori* (*H.pylori*) eradication is crucial, since even minor inaccuracies can have major impact. To determine the cut-off point for ^13^C-urea breath test (^13^C-UBT) and to assess if it can be further optimized by serologic testing, mathematic modeling, histopathology and serologic validation were applied. A finite mixture model (FMM) was developed in 21,857 subjects, and an independent validation by modified Giemsa staining was conducted in 300 selected subjects. *H.pylori* status was determined using recomLine *H.pylori* assay in 2,113 subjects with a borderline ^13^C-UBT results. The delta over baseline-value (DOB) of 3.8 was an optimal cut-off point by a FMM in modelling dataset, which was further validated as the most appropriate cut-off point by Giemsa staining (sensitivity = 94.53%, specificity = 92.93%). In the borderline population, 1,468 subjects were determined as *H.pylori* positive by recomLine (69.5%). A significant correlation between the number of positive *H.pylori* serum responses and DOB value was found (r_s_ = 0.217, *P* < 0.001). A mathematical approach such as FMM might be an alternative measure in optimizing the cut-off point for ^13^C-UBT in community-based studies, and a second method to determine *H.pylori* status for subjects with borderline value of ^13^C-UBT was necessary and recommended.

## Introduction

Gastric cancer (GC) is a global public health burden with an annual incidence of about one million, of which 42% occurred in China^1, 2^. The *Helicobacter pylori* (*H.pylori*) is known as the strongest risk factor for GC^3^. Accumulated evidences from our two intervention trials in Linqu County, a high-risk area of GC in Northern China with an exceptionally high prevalence of *H.pylori* infection (72% for adults) revealed that *H.pylori* eradication could reduce the risk of GC and its precursors^4, 5^. In 2011, we launched a randomized controlled intervention trial in Linqu County to prevent GC by eradication of *H.pylori* (Linqu Trial) in cooperation with the International Digestive Cancer Alliance and Technical University of Munich. This trial aimed at assessing if eradication of *H.pylori* can effectively reduce GC incidence among 184,786 adults, in which ^13^C-urea breath test (^13^C-UBT) was applied to determine *H.pylori* status^6^.

The ^13^C-UBT is considered one of the most proper diagnostic measures for *H.pylori* infection screening because of its non-invasiveness and accuracy^7^. However, the dose of ^13^C-urea and types of equipment are directly related to the results of ^13^C-UBT. Therefore, the cut-off point should be adjusted in different populations^8–10^, especially in large intervention trials. In addition, the existence of a “gray zone” in ^13^C-UBT, which comprises approximately 1~2% of the test population and leads to misclassification of *H.pylori* infection^11, 12^, also urges a more precise cut-off point in a large population-based study.

Ideally, an endoscopy based bacterial culture or histologic diagnosis should be applied as a gold standard to determine the cut-off point of ^13^C-UBT^13^. But this approach was not appropriate for such a large-scale community-based trial due to its invasiveness. The finite mixture model (FMM) has been applied in calculating the cut-off point of diagnostic tests including ^13^C-UBT recently^14^, suggesting mathematical tools could serve as alternative measures for optimising the cut-off point of ^13^C-UBT in our large trial. In addition, the recomLine *H.pylori* immunoglobulin G (IgG) assay, as a novel serological method for detecting *H.pylori* infection with high sensitivity (97.6%) and specificity (96.2%)^15^, might be useful for further optimizing a cut-off point and assessing *H.pylori* status in subjects within the “gray zone” of ^13^C-UBT.

In this study, we applied a FMM based on the expectation maximization (EM) algorithm to establish an optimal cut-off point of ^13^C-UBT in a modelling dataset randomly selected by clusters (first 50 out of 980 villages participated in Linqu Trial) from the trial participants. Then, we validated it taking modified Giemsa stain as reference. Subjects within the “gray zone” of ^13^C-UBT were also selected to assess if the cut-off value can be further optimized by recomLine assay.

## Results

A total of 21,857 subjects (9,360 males and 12,497 females) were selected as the modelling dataset, with the mean age of 45.6 ± 8.3 years. The mean age of validating subjects (44.5 ± 4.8 years) was significantly younger than that of the modelling population (*P* < 0.001). The distribution of alcohol drinking was also statistically different between the modelling population and validating subjects (*P* < 0.001). No statistical difference was found in smoking among modeling, validating subjects and borderline populations, while the frequency of male (47.6%, *P* < 0.001) or alcohol drinking (28.5%, *P* = 0.009) was significantly higher in the borderline subjects (Table 1).

**Table 1:** Baseline characteristics of participants in different study populations.

Characteristics	Modelling population n = 21,857	Validating subjects n = 300		Borderline subjects n = 2,113	
	N (%)	N (%)	*P* ^*^	N (%)	*P* ^*^
Age(Mean ± SD)	45.6 ± 8.3	44.5 ± 4.8	<0.001**	45.7 ± 8.2	0.624**
Sex			0.600		<0.001
Male	9,360(42.8)	133(44.3)		1,009 (47.8)	
Female	12,497(57.2)	167(55.7)		1,104 (52.2)	
Smoking			0.887		0.284
No	17,102(78.2)	222(74.0)		1,632 (77.2)	
Yes	4,755(21.8)	63(21.0)		481 (22.8)	
Missing	0(0.0)	15(5.0)		0(0.0)	
Drinking			<0.001		0.009
No	16,108(73.7)	238(79.3)		1,502 (71.1)	
Yes	5,749(26.3)	47(15.7)		611 (28.9)	
Missing	0(0.0)	15(5.0)		0(0.0)	

^*^Pearson’s χ^2^ test compared to the modelling population without missing values.

^**^Student’s t-test.

**Figure 1:**
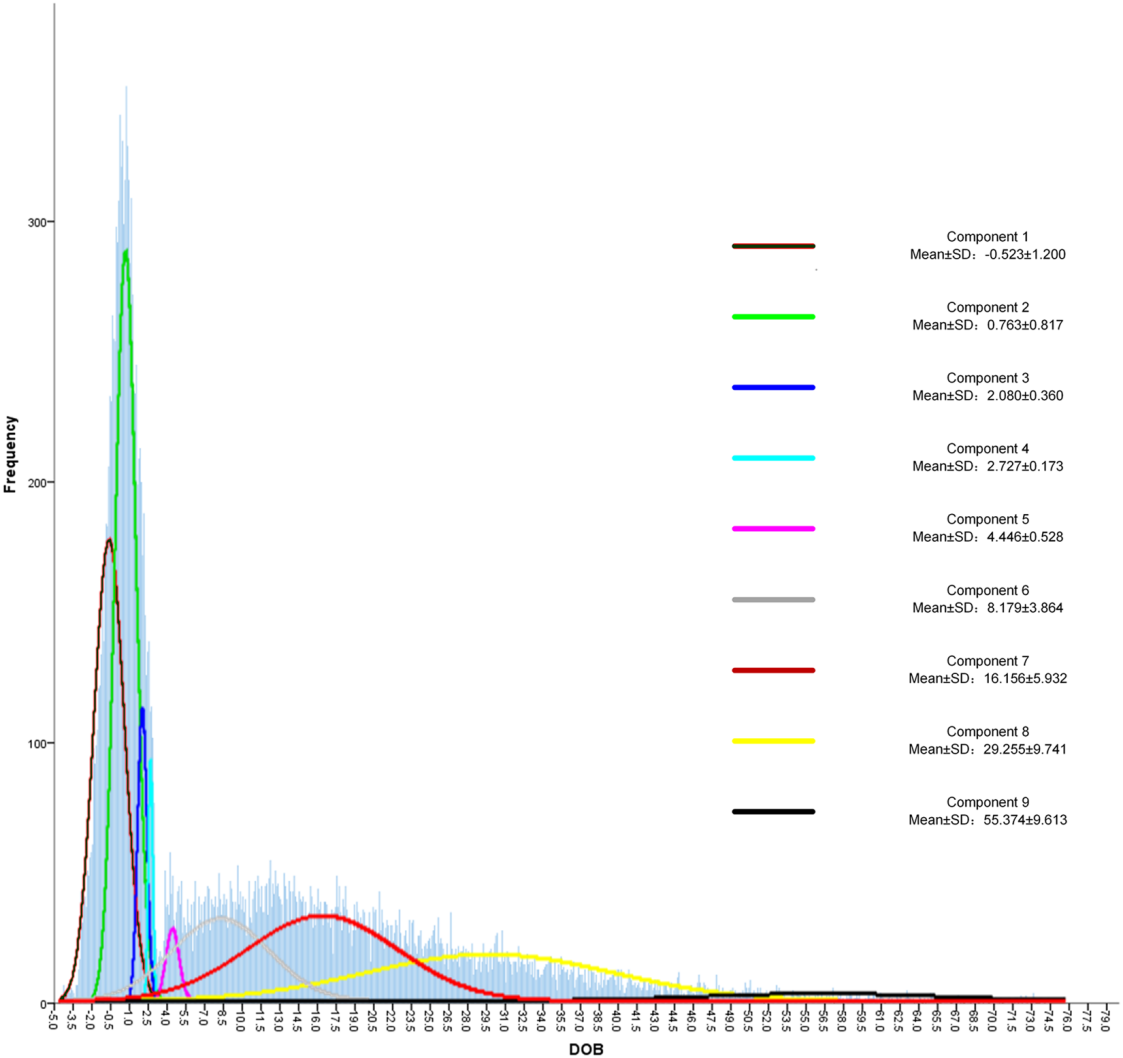
Distribution of DOB value with density line of 9 components modeled by finite mixture model in the modelling population.

The minimum, median and maximum values of DOB in the modelling population were −395.76, 3.05 and 312.38, respectively, while the quartile range was 17.33, indicating outliers might exist. Most of the DOB values (99%) were located in the interval from −2.86 to 64.73, the accumulate percentages of subjects with DOB < 3.0 and 3.0 ≤ DOB < 4.0 were 49.92% and 1.2% respectively. The distribution was more discrete in DOB ≥ 4.0 compared to subjects with DOB < 3.0 (Fig. 1).

A total of 21,639 subjects were finally analyzed after excluding the outliers by P_0.5_ (DOB = −4.49) and P_99.5_ (DOB = 74.42) to fit the finite mixture model (FMM). Based on the fact that this population should be classified into at least 2 groups, namely, *H.pylori* positive and *H.pylori* negative, the K components FMM was generated with initial K = 2, increasing 1 more component each step. By comparing Akaike’s Information Criterion (AIC) and the Bayesian Information Criterion (BIC) between different models, the model containing 9 components with the smallest AIC (149487.2) and BIC (149547.9) was chosen as the best fitting model (Fig. 1 and Supplementary Table 1).

The means and SDs of the 9 components were presented in Supplementary Table 2. Previous studies applying the same ^13^C-UBT procedure as the Linqu Trial usually took DOB = 4.0^10^ as a cut-off point. Thus, the 9 subgroups were assigned as putative *H.pylori* negative group (subgroup 1, 2, 3 and 4) and putative *H.pylori* positive group (subgroup 5, 6, 7, 8 and 9). The putative sensitivities and specificities were then calculated under a set of cut-off points. The cut-off value of DOB = 3.1 yielded the highest Youden’s index whereas the specificity reached 99.99% with a sensitivity of 95.92% when choosing DOB = 3.8 as cut-off value (Table 2).

**Table 2:** Putative sensitivity and specificity of cut-off points by finite mixture model.

Cut-off	Sensitivity (%)	Specificity (%)	Youden’s index
2.4	97.99	94.04	0.9203
2.5	97.89	95.11	0.9300
2.6	97.79	96.19	0.9398
2.7	97.68	97.30	0.9498
2.8	97.57	98.33	0.9590
2.9	97.45	99.10	0.9655
3.0	97.33	99.56	0.9689
**3.1**	**97.20**	**99.78**	**0.9698**
3.2	97.06	99.87	0.9693
3.3	96.91	99.92	0.9683
3.4	96.75	99.95	0.9670
3.5	96.58	99.96	0.9654
3.6	96.38	99.97	0.9635
3.7	96.16	99.98	0.9614
**3.8**	**95.92**	**99.99**	**0.9591**
3.9	95.65	99.99	0.9564
4.0	95.35	99.99	0.9534
4.1	95.03	99.99	0.9502
4.2	94.67	99.99	0.9466

We further assessed these cut-off values in 300 validation subjects applying modified Giemsa staining as a gold standard, among which, 201 were *H.pylori* positive and 99 were *H.pylori* negative. ROC curve analysis found that the area under curve (AUC) was 0.960 (95%CI: 0.932–0.989) and determined DOB = 3.8 as the best cut-off point, with a sensitivity of 94.53% (190/201), a specificity of 92.93% (92/99), and a kappa value of 0.866 (*P* < 0.001). In contrast, the sensitivity and specificity were 94.53% (190/201) and 89.90% (89/99) respectively, taking a DOB = 3.1 as the cut-off value (Table 3 and Supplementary Figure 1).

**Table 3:** Sensitivity and specificity of ^13^C-UBT in validating subjects.

^13^C-UBT		Giemsa (N, %)		Total	Kappa	*P*
		Positive	Negative			
Cut-off = 3.1	Positive	190(94.53)	10(10.10)	200	0.842	<0.001
	Negative	11(5.47)	89(89.90)	100		
Cut-off = 3.8	Positive	190(94.53)	7(7.07)	197	0.866	<0.001
	Negative	11(5.47)	92(92.93)	103		
Total		201(100.00)	99(100.00)	300		

Furthermore, we evaluated the consistency of *H.pylori* infection results between recomLine *H.pylori* test with Giemsa staining and ^13^C-UBT in the validation subjects. The recomLine *H.pylori* test gained a sensitivity of 98.01% (197/201) and specificity of 81.82% (81/99) with a kappa value of 0.828 (*P* < 0.001) taking Giemsa staining as a reference method. In contrast to ^13^C-UBT, the true positive rate of recomLine test was 94.92% (187/197), the true negative rate was 72.82% (75/103), and the agreement rate was 87.33% (262/300) with a kappa value of 0.707 (*P* < 0.001) (Table 4).

**Table 4:** Consistency of *H.pylori* test results among diagnostic methods.

Reference Methods		recomLine (N,%)		Total	Kappa	*P*
		Positive	Negative			
Giemsa	Positive	197(98.01)	4(1.99)	201(100.00)	0.828	<0.001
	Negative	18(18.18)	81(81.82)	99(100.00)		
^13^C-UBT	Positive	187(94.92)	10(5.08)	197(100.00)	0.707	<0.001
	Negative	28(27.18)	75(72.82)	103(100.00)		
Total		215	85	300		

We were also interested to check if the cut-off point can be further optimized by serology using the recomLine *H.pylori* test, as well as the correlation between the distribution of DOB value and seroprevalence of *H.pylori* in the borderline population. A total of 2,113 subjects with DOB values ranged from 2.5 to 4.0 were further selected, among which 1,646 subjects with DOB value below 3.8 were assigned as negative borderline group in contrast to 467 subjects of positive borderline with DOB ≥ 3.8. By recomLine test, the seroprevalence of *H.pylori* IgG antibody was 69.5% in this borderline population, and was significantly higher in positive borderline group (3.8 ≤ DOB < 4.0) compared to negative group (83.1% vs. 65.5%, *P* < 0.001). A weak correlation between the number of positive *H.pylori* serum responses and DOB value was found with statistical significance (r_s_ = 0.217, *P* < 0.001). Similarly, seropositivities of *H.pylori* specific antibodies CagA (73.02% vs. 52.86%, *P* < 0.001), VacA (30.84% vs. 17.13%, *P* < 0.001), GorEL (52.03% vs. 34.14%, *P* < 0.001), UreA (25.91% vs. 19.74%, *P* = 0.004), HcpC (58.46% vs. 36.03%, *P* < 0.001), and gGT (49.04% vs. 33.29%, *P* < 0.001) were significantly higher in the positive borderline group compared to the negative borderline group (Table 5 and Supplementary Figure 2). However, further subgroup analysis in the negative borderline subjects (2.5 ≤ DOB < 3.8) did not observe a statistical association between *H.pylori* seropositivities and DOB values (*P* = 0.118).

**Table 5:** Seropositivities for *H.pylori* specific antibodies in borderline subjects with different DOB value.

	Total (N, %)	DOB Groups (N, %)		*P*
		Negative borderline (2.5 ≤ DOB < 3.8)	Positive borderline (3.8 ≤ DOB < 4.0)	
CagA				<0.001
Negative	902(42.69)	776(47.14)	126(26.98)	
Positive	1211(57.31)	870(52.86)	341(73.02)	
VacA				<0.001
Negative	1687(79.84)	1364(82.87)	323(69.16)	
Positive	426(20.16)	282(17.13)	144(30.84)	
GroEL				<0.001
Negative	1308(61.90)	1084(65.86)	224(47.97)	
Positive	805(38.10)	562(34.14)	243(52.03)	
UreA				0.004
Negative	1667(78.89)	1321(80.26)	346(74.09)	
Positive	446(21.11)	325(19.74)	121(25.91)	
HcpC				<0.001
Negative	1247(59.02)	1053(63.97)	194(41.54)	
Positive	866(40.98)	593(36.03)	273(58.46)	
gGT				<0.001
Negative	1336(63.23)	1098(66.71)	238(50.96)	
Positive	777(36.77)	548(33.29)	229(49.04)	

DOB, Delta over baseline-value.

.

## Discussion

Based upon the large intervention trial, we established an optimal cut-off point for ^13^C-UBT and explored the borderline of DOB in this high risk population. Findings in this study provided the basic data for our large trial.

The ^13^C-UBT is one of the most reliable methods detecting the presence of an active *H.pylori* infection^16^. The non-invasiveness of the ^13^C-UBT resulting in a high acceptance in patients makes it widely adopted in clinical and epidemiological practices. However, the results of ^13^C-UBT vary with populations, doses of ^13^C-urea, and types of equipment, therefore, the cut-off point should be adjusted in different populations^8–10, 17, 18^. The recommended cut-off values of ^13^C-UBT fluctuate between 2.5 and 4.0 while the dose of ^13^C-urea changes from 75 mg to 100 mg^10, 13, 19, 20^. In the Linqu Trial, a widely accepted dosage of 75 mg ^13^C-urea (Min.99 atom % ^13^C) in Asian populations was applied in ^13^C-UBT, and the suggested cut-off point is DOB = 4.0^10^. In this large scale study, a small proportion of misclassification would affect a considerable number of subjects. Thus, in the Linqu Trial aimed at approximately 200,000 participants, it is necessary to optimize the cut-off point of ^13^C-UBT.

In this study, we applied the EM algorithm based Gaussian FMM to fit the DOB distribution in modelling dataset and established optimal cut-off points. The FMM provides us a mathematical approach to fit complicated distribution by a set of simple distributions, allowing us to assess the cut-off point based on the fitting density curve, especially in studies lacking a gold standard. Du *et al*. have established a cut-off point for ^13^C-UBT in children^14^ via this model. In our study, we chose a more conservative cut-off point (DOB = 3.8) to achieve a high specificity instead of a cut-off point (DOB = 3.1) with the highest Youden’s index after model fitting. By this means, we aimed at avoiding unnecessary antibiotic treatment in *H.pylori* negative subjects.

However, the model oriented cut-off point would be more reliable if it was validated independently by a gold standard. Further validation in 300 independent subjects selected from the same trial participants supported the optimal cut-off point as DOB = 3.8. The sensitivity (94.53%) was in line with those of previous studies while the specificity (92.93%) was slightly lower than them^21^.

The lower specificity in the current study was probably attributed to the misclassification caused when only Giemsa staining was applied as the gold standard, whereas other studies jointly used two or three methods as references, such as rapid urease test, microbiological culture and histology^22^. Giemsa staining is widely applied as a gold method in diagnositc test for *H.pylori* infection^23^. However, the accuracy is affected by the density of *H.pylori* in the stomach, sites of biopsy and the procedure of staining. Although we had varified Giemsa negative subjects in hematoxylin-eosin staining slides from the same biopsy site in this study, there were still risks of false negative diagnosis of *H.pylori* due to a limited number of biopsy sites.

We were also interested in whether the cut-off point can be further optimized by serology using the recomLine *H.pylori* test. The recomLine *H.pylori* IgG assay is a rapid line immune assay, which allows the identification of specific antibody responses against distinct *H.pylori* antigens. By contrasting to histology diagnosis, the recomLine assay was reported with a sensitivity and specificity of 97.6 and 96.4% respectively^15^. In this study, we first evaluated the agreement between recomLine *H.pylori* test and Giemsa staining or ^13^C-UBT, and found that the recomLine test gained a sensitivity of 98.01% taking Giemsa staining as a gold method, yet the specificity was 81.82%. The high sensitivity indicates this recomLine test could be an initial screening tool for *H.pylori* infection in population-based studies. The positive results obtained by recomLine assay in some Giemsa-negative cases could result from discontinous colonization and thus false negative Giemsa staining, or might be a consequence of past exposure to *H.pylori* in this high-risk area of GC.

We further investigated the recomLine score and specific antibody responses of these 18 discrepant subjects, which are recomLine positive but Giemsa and ^13^C-UBT negative. The total socre of recomLine test was significantly lower in these cases comparing with the 187 triple-positive subjects of recomLine, Giemsa and ^13^C-UBT (3.79 ± 1.41 vs. 4.88 ± 1.67, *P* = 0.007). Furthermore, seropositivities of GorEL (16.67% vs. 67.91%, P < 0.001) and gGT (16.67% vs. 59.36%, *P* = 0.001) were significantly lower in the discrepant cases comparing with the triple-positive subjects (Supplementary Table 3). While such lower antibody responses are often observed after eradication, studies with a larger sample size and detailed information of past exposure to *H.pylori* are needed to further explore this.

Our serologic results on borderline population further confirmed our findings. We found that seropositivity of *H.pylori* was significantly more frequent in the positive borderline population compared to the negative borderline. Although no statistical association between the distribution of DOB value and seropositivity of *H.pylori* was found, a significant correlation between the number of positive *H.pylori* serum responses and DOB value was observed. In addition to the existence of “gray zone”, past exposure to *H.pylori* might account for the high seropositivity in subjects with negative borderline results, since *H.pylori* CagA antibody persisted even many years after eradication.

Beside the optimal cut-off point, the “gray zone” in ^13^C-UBT also influences the determination of *H.pylori* status in epidemiological studies. Affected by natural variations of exhaled ^13^CO_2_ and detection accuracy of gas isotopic ratio mass spectrometer (GIRMS), approximately 1% to 2% of test subjects present with a DOB value in the “gray zone” around the cut-off value of ^13^C-UBT^24^. In this study, the “gray zone” was defined as ranging from 2.5 to 4.0. For those subjects, more attention should be paid to discover any symptoms of gastric diseases, which should undergo further examination or treatment. The high seropositivity in subjects with DOB around 2.5 suggested that the interval of the “gray zone” requires further exploration, and a second *H.pylori* diagnostic test must be applied in this “gray zone” population.

Several strengths were shown in this study. Firstly, we established an optimal cut-off point of ^13^C-UBT in a large population at high risk of GC via FMM with histological validation. To our knowledge, this is the first study to optimize the cut-off point of ^13^C-UBT in such large population jointly by mathematical and histological means. Secondly, in subjects with histological information, diagnostic values of ^13^C-UBT and recomLine *H.pylori* test were further assessed, and a higher sensitivity was observed for the recomLine *H.pylori* test, which suggested that this novel serological test could serve as an initial and complementary tool in *H.pylori* screening in such high risk population. Thirdly, our findings supported the existence of “gray zone” and suggested that a second measure should be implicated in those subjects after ^13^C-UBT.

Our study also has some limitations. The sample size of validating subjects was limited, especially for subjects with DOB around the cut-off point. Thus, studies with a larger sample size are still needed for a more thorough characterization of the “gray zone” population, and to determine the delimiters/borders of such “gray zone”. To avoid interfering with the real-word setting of the Linqu Trial, subjects for the validation set were from the overlapping population of the Linqu Trial and an endoscopy-based GC screening program. Consequently, the sample size of this validation cohort is relatively small and some demographic factors, such as age, gender and alcohol consumption, were different from the modeling set. Although no previous studies reported the correlation between these demographic factors with cut-off values of ^13^C-UBT in adults, a validation with larger sample size and completely matched population is warranted to drawn a firm conclusion. Moreover, limited by information of past exposure to *H.pylori* and biopsies taken from validating subjects, discordant results between all the three tests could not be further explored. To address this issue, further assessment should be conducted in a larger population with complete information on *H.pylori* exposure and anti-*H.pylori* treatment, jointly using histopathological and bacterial culture as references.

In conclusion, FMM might be an alternative measure in optimizing cut-off point for ^13^C-UBT in community-based studies. A DOB value of 3.8 was determined and validated as an optimal cut-off point for ^13^C-UBT in the Linqu Trial. The recomLine test could be an initial screening tool for *H.pylori* infection in population-based studies. For the “gray zone” population, a second measure as a complement for ^13^C-UBT is necessary and recommended in determining *H.pylori* status.

## Methods

### Study population

This study was based upon a large community-based intervention trial conducted in Linqu County (Linqu Trial, registered as ChiCTR-TRC-10000979)^6^. The Linqu Trial enrolled 184,786 residents aged 25~54 years from 980 villages. Participants with any of the following conditions were excluded from the trial at the enrollment as described previously^6^, i.e., peptic ulcers, serious medical conditions, undergoing active treatment for cancer, currently or previously on antibiotic therapy for *H.pylori* infection, history of congestive heart failure, respiratory failure, stroke, seizures, pregnancy, and mental or psychiatric illness. After a ^13^C-UBT for determination of *H.pylori* status at baseline, *H.pylori* positive participants were allocated into two groups using cluster randomization by village, being given either a 10-day bismuth-based quadruple anti-*H.pylori* treatment or look-alike placebos together with single dosage of 20 mg omeprazole and 300 mg bismuth citrate. Information on demographic characteristics, medical history, dietary, and cigarette and alcohol consumption was collected by a structured questionnaire.

In the current study, 21,857 subjects in the beginning 50 villages of the trial population were selected as modelling dataset for finite mixture model (FMM). According to previous publications, most cut-off values ranged from 2.5 to 4.0 when the dose of ^13^C-urea was 75 mg in ^13^C-UBT^10, 13, 19, 20^. Hereby, all the 2,113 with DOB values ranged from 2.5 to 4.0 out of 103,000 subjects completed ^13^C-UBT at the end of June, 2012 were further selected to explore the boundary of the “gray zone” of ^13^C-UBT.

To validate the FMM oriented cut-off points, an independent validation was conducted in 300 cancer-free subjects who participated in both an endoscopy-based GC screening program and the Linqu Trial. Endoscopy based biopsies and baseline ^13^C-UBT screening were taken within 6 months prior to anti-*H.pylori* intervention of the Linqu Trial. For these subjects, *H.pylori* status was confirmed by modified Giemsa staining.

### Ethic statement

All experiments were performed in accordance with relevant guidelines and regulations. This study was approved by the Institutional Review Board of Peking University Cancer Hospital & Institute and collaborating institutions, and an informed written consent was obtained from each participant.

### Methodology of ^13^C-UBT

As described previously^6^, each subject was requested to swallow a pill containing 75 mg ^13^C-urea (Min.99 atom % ^13^C, Campro Scientific, Germany) with 20 ml of water after a baseline breath samples (T_0_) were collected in the morning. Exhaled air was collected in sampling tubes 30 minutes later (T_30_). The concentration of ^13^CO_2_ was determined by gas isotopic ratio mass spectrometer (ZHP-2001, KYKY TECHNOLOGY Co., LTD, Beijing, China), the difference between T_30_ and T_0_ was expressed by and delta over baseline-value (DOB).

### Giemsa stain

The modified Giemsa stain was performed using a commercial kit (Beijing Leagene Biotechnology Co., China). Briefly, after de-paraffinization and rehydration, tissue sections were immersed in Giemsa working solution at room temperature for 30 minutes, and then dehydrated. *H.pylori* was stained blue and the bacterial density was graded by two trained investigators independently as described by Gray *et al*.^25^.

### Blood sample collection

An 8-mL blood sample was collected from each subject by a BD Vacutainer PPT™ Plasma Preparation Tube and a K_2_-EDTA tube (BD, NJ, USA). Once the whole blood sample was collected, the tube was gently inverted 8~10 times, and then centrifuged at 1,100 RCF for 15 minutes at room temperature. The resulting undiluted EDTA plasma was separated into vials and was frozen immediately at −20 °C and stored at −70 °C. The blood cells were frozen immediately at −20 °C for further utilization.

### recomLine *H.pylori* IgG analysis

The recomLine *H.pylori* IgG is a line immunoassay (Mikrogen, Germany) contains highly purified recombinant *H.pylori* antigens (CagA, VacA, GroEL, UreA, HcpC and gGT) immobilized on nitrocellulose membrane strips^15^. As described previously^15^, test strips were incubated with the diluted plasma sample, after washing, strips were incubated with anti-human immune globulin antibodies coupled to horseradish peroxidase. Followed three washing steps removing unbound conjugate antibodies, bound antibodies were detected by a peroxidase based staining reaction leading to a dark band appearing on the strip at the corresponding antigen lane.

The test result was determined according to the total scores of the individual band. A subject was considered to be positive if the total score ≥2.0. The score of individual antigens was assigned as follows: 2 points for CagA, VacA, and GroEL, 1 point for UreA, HcpC, and gGT. Thus, the total score of the assay ranges from 0 (all negative) to 9 (all positive).

### Statistical Methods

A Gaussian FMM with K components based on expectation maximization (EM) algorithm was applied to fit the DOB distribution of modelling dataset and to establish the optimal cut-off point. By the EM algorithm, parameters in each model were estimated by maximum likelihood. Models with increasing numbers of components were fitted to determine the best model to cluster the DOB values. The Akaike’s Information Criterion (AIC) and the Bayesian Information Criterion (BIC) were used to indicate the best fitted model^26^. The modelling procedure was conducted using the “mixtools” package (Version 0.4.6, http://cran.r-project.org/web/packages/mixtools/index. html) in R program as described^27^. With the estimated means, standard deviations (SDs) and proportions for each component of the best-fitted model, putative sensitivities, specificities and Youden’s indexes were calculated under corresponding DOB values.

The receiver operating characteristic (ROC) curve was employed to evaluate the optimal cut-off point of ^13^C-UBT in the validating population. The t-test or χ^2^ test was used to examine the differences of characteristics between different study populations, as well as the association between *H. pylori* seropositivity and DOB group in ^13^C-UBT borderline subjects. Correlation between the number of positive *H. pylori* serum responses and DOB value was tested by Spearman’s correlation analysis. All statistical analyses were conducted by Statistical Analysis System software (version 9.2; SAS Institute, Cary, NC), a *P* value of <0.05 was considered significant and all statistical tests were two sided.

## Electronic supplementary material

Supplementary informations
