# Supplementary material for: Cut-off optimization for 13C-urea breath test in a community-based trial by mathematic, histology and serology approach
Source: Sci Rep. 2017 May 18;7:2072. doi: 10.1038/s41598-017-02180-7 (PMC5437005; doi:10.1038/s41598-017-02180-7)
Supplement: Supplementary file 1 — Supplementary informations [file 41598_2017_2180_MOESM1_ESM.pdf]

Cut-off optimization for ^{13}C-urea breath test in a community-based trial by mathematic, histology and serology approach

Zhe-Xuan Li[1†], Lei-Lei Huang[1†], Cong Liu[1], Luca Formichella[2], Yang Zhang[1], Yu-Mei Wang[1], Lian Zhang[1], Jun-Ling Ma[1], Wei-Dong Liu[3], Kurt Ulm[2], Jian-Xi Wang[3], Lei Zhang[1], Monther Bajbouj[2], Ming Li[3], Michael Vieth[4], Michael Quante[2], Tong Zhou[1], Le-Hua Wang[3], Stepan Suchanek[5], Erwin Soutschek[6], Roland Schmid[2], Meinhard Classen[2,7], Wei-Cheng You[1], Markus Gerhard[2,8], and Kai-Feng Pan[1]

[1]Key Laboratory of Carcinogenesis and Translational Research (Ministry of Education/Beijing), Department of Cancer Epidemiology, Peking University Cancer Hospital & Institute, 52 Fu-cheng Road, Hai-dian District, Beijing 100142, China. [2]Technische Universität München, Klinikum rechts der Isar, Trogerstr. 30, 81675 Munich, Germany; [3]Healthy Bureau of Linqu County, Shandong, China; [4]Institute of Pathology, Klinikum Bayreuth, Preuschwitzer Str. 101, 95445 Bayreuth, Germany; [5]Charles University, Central Military Hospital Prague, Ovocný trh 3-5, Prague, 11636, Czech Republic; [6]Mikrogen GmbH, Floriansbogen 2-4, Neuried, Munich 82061, Germany, [7]International Digestive Cancer Alliance, 81541 Munich, Germany; [8]German Centre of Infection Research, partner site Munich, Germany.

Correspondence: Kai-Feng Pan, Key Laboratory of Carcinogenesis and Translational Research (Ministry of Education/Beijing), Department of Cancer Epidemiology, Peking University Cancer Hospital & Institute, 52 Fu-cheng Road, Hai-dian District, Beijing 100142, China; Email: [pankaifeng2002@yahoo.com]; or Markus Gerhard, Institute of Medical Microbiology, Immunology and Hygiene, Technische Universität München, Trogerstr. 30, 81675 Munich, Germany. E-mail: [Markus.Gerhard@tum.de].

[†] Zhe-Xuan Li[1] and Lei-Lei Huang[1] contributed equally to this work.

Running title: Cut-off point of ^{13}C-UBT for *H.pylori*

Supplementary Table 1. Selection of the Best Fitting Finite Mixture Model

No. of components	No. of observations	Log Likelihood	AIC	BIC
2	21639	-76175.83	152361.7	152373.3
3	21639	-75063.10	150142.2	150160.9
4	21639	-75049.50	150121.0	150146.7
5	21639	-74875.03	149778.1	149810.8
6	21639	-74834.09	149702.2	149741.9
7	21639	-74776.57	149593.1	149639.9
8	21639	-74750.72	149547.4	149601.2
**9**	21639	**-74717.59**	**149487.2**	**149547.9**
10	21639	-74785.64	149629.3	149697.0

AIC, Akaike's Information Criterion; BIC, Bayesian Information Criterion

Supplementary Table 2. Parameters of the 9 Components in the Best Fitting Model

Subgroup	Weight of component	Mean±SD
1	0.205	-0.523±1.200
2	0.227	0.763±0.817
3	0.039	2.080±0.360
4	0.016	2.727±0.173
5	0.014	4.446±0.528
6	0.118	8.179±3.864
7	0.187	16.156±5.932
8	0.168	29.255±9.741
9	0.026	55.374±9.613

Supplementary Table 3. Seropositivities for *H.pylori* antibodies in recomLine positive subjects with different Giemsa and ^{13}C-UBT results

	recomLine positive but Giemsa and ^{13}C-UBT negative N=18	Triple-positive of recomLine, Giemsa and ^{13}C-UBT N=187	*P*
CagA (%)			0.533
Negative	3(16.67)	28(14.97)	
Positive	15(83.33)	159(85.03)	
VacA (%)			0.106
Negative	8(44.44)	117(62.57)	
Positive	10(55.56)	70(37.43)	
GroEL (%)			<0.001
Negative	15(83.33)	60(32.09)	
Positive	3(16.67)	127(67.91)	
UreA (%)			0.127
Negative	13(72.22)	160(85.56)	
Positive	5(27.78)	27(14.44)	
HcpC (%)			0.313
Negative	10(55.56)	87(46.52)	
Positive	8(44.44)	100(53.48)	
gGT (%)			0.001
Negative	15(83.33)	76(40.64)	
Positive	3(16.67)	111(59.36)	

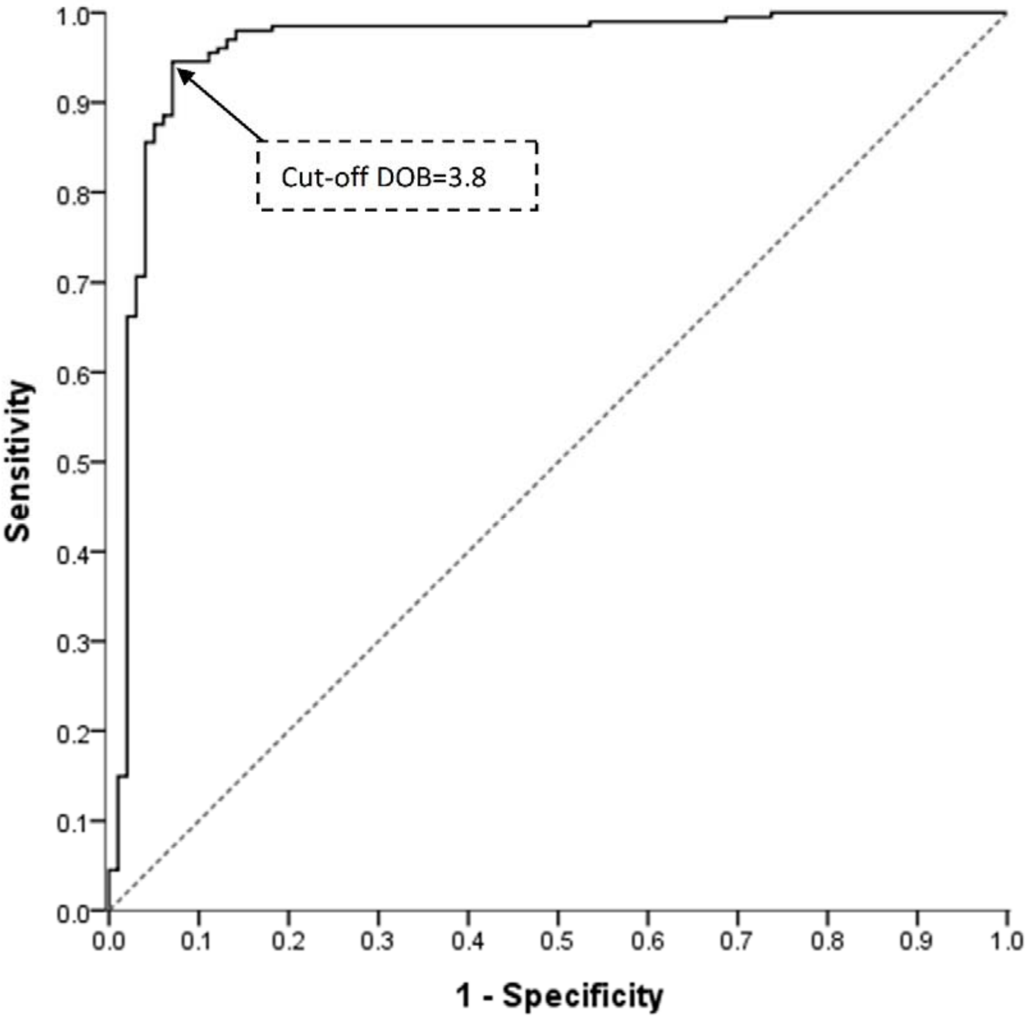

Supplementary Figure 1 Receiver operating characteristics curve analysis of ^{13}C-UBT in validating subjects taking Giemsa stain as a gold standard.

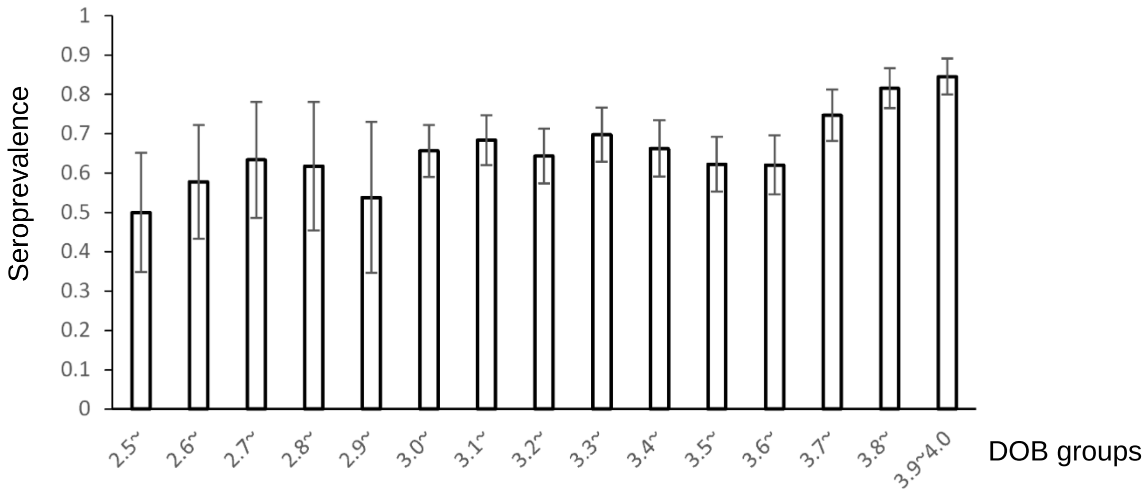

Supplementary Figure 2 The seroprevalence of *H.pylori* by recomLine in borderline subjects with different DOB value.

DOB, Delta over baseline-value.

DOB groups were generated from DOB values with 0.1 change:

2.5~ (2.5≤DOB<2.6), 2.6~ (2.6≤DOB<2.7), 2.7~ (2.7≤DOB<2.8),
2.8~ (2.8≤DOB<2.9), 2.9~ (2.9≤DOB<3.0), 3.0~ (3.0≤DOB<3.1),
3.1~ (3.1≤DOB<3.2), 3.2~ (3.2≤DOB<3.3), 3.3~ (3.3≤DOB<3.4),
3.4~ (3.4≤DOB<3.5), 3.5~ (3.5≤DOB<3.6), 3.6~ (3.6≤DOB<3.7),
3.7~ (3.7≤DOB<3.8), 3.8~ (3.8≤DOB<3.9), 3.9~4.0 (3.9≤DOB<4.0).
